# Using wearable and nearable devices in telerehabilitation for COPD: a scoping review of digital endpoints in home-based programs

**DOI:** 10.3389/fdgth.2026.1698019

**Published:** 2026-02-03

**Authors:** Stephanie Zawada, Louis Faust, Caden Collins, Moein Enayati, Roberto Benzo, Emma Fortune

**Affiliations:** 1 Robert D. and Patricia E. Kern Center for the Science of Health Care Delivery, Mayo Clinic, Rochester, MN, United States; 2 Division of Health Care Delivery Research, Mayo Clinic, Rochester, MN, United States; 3 Department of Neurology, Mayo Clinic, Scottsdale, AZ, United States; 4 Mayo Clinic Platform, Rochester, MN, United States; 5 Division of Pulmonary and Critical Care Medicine, Mayo Clinic Mindful Breathing Laboratory, Rochester, MN, United States

**Keywords:** remote monitoring, COPD, wearable sensors, digital endpoint, pulmonary rehabilitation

## Abstract

**Introduction:**

Despite its demonstrated effectiveness at improving outcomes, pulmonary rehabilitation (PR) for chronic obstructive pulmonary disease (COPD) is underutilized. Sensor-generated data from wearable devices have the potential to mitigate this challenge by generating digital endpoints that provide insights into patient behaviors at home; however, there is no consensus on how to measure home-based PR (HBPR) outcomes with these tools. This review aims to describe (1) the most frequent digital endpoints used in HBPR studies and (2) the devices used to capture these endpoints, summarizing gaps in their applications to HBPR for COPD patients.

**Methods:**

We completed a scoping review using the PRISMA checklist across databases (Web of Science, Scopus, and OVID) from January 1, 2005 to June 1, 2025. We included peer-reviewed articles on HBPR for COPD, excluding reviews, commentaries/editorials, poster abstracts, and conference proceedings. Eligible articles included cohort studies and clinical trials of adult patients (age ≥ 18 years) with COPD participating in HBPR that include one or more digital endpoints.

**Results:**

Among eligible articles (*n* = 218), 13 (6.0%) met inclusion criteria, the majority of which were published after 2020 (61.5%). Most studies enrolled fewer than 100 COPD patients (76.9%) for an average monitoring period of 12.5 weeks. Activity trackers were the most commonly used device (46.2%) to capture data. The most frequently used digital endpoints were step count (84.6%), time spent active (38.5%), and time spent sedentary (30.8%). Two study designs were used: randomized controlled trial (76.9%) and observational cohort. Study designs were heterogenous with more than one-third (38.5%) presenting a lack of statistically significant results.

**Discussion:**

Although we identified analogous digital endpoints in some studies, dissimilar methods and study designs remain barriers to synthesizing results generated from HBPR programs for COPD. Wearable devices have the potential to build novel PR models, but more work is needed to translate real-world data into clinically meaningful measures. Future research should elucidate which participants would benefit most from and complete HBPR to build an evidence base for the validation of HBPR-relevant digital endpoints, particularly those derived from less common sources like cardiovascular and sleep measures.

## 1 Introduction

Chronic obstructive pulmonary disease (COPD) is a leading cause of death worldwide, and its prevalence is projected to increase by 2030 (1, 2). Exacerbations of COPD, characterized by worsening of chronic breathing problems, are a major cause of hospitalization and death. In addition, these episodes place significant stress on the heart, contributing to the estimated 60% of COPD-related deaths attributed to cardiovascular disease (3). Considering that 65% of patients with COPD are overweight or obese, the need for scalable, effective prevention strategies to lower cardiopulmonary risk factors, like sedentary behavior, in this population is urgent (3). Pulmonary rehabilitation (PR) is one such approach to increase physical activity, reduce shortness of breath, and enhance mood in patients with COPD (4, 5).

PR is a well-established treatment, effective for improving outcomes and quality-of-life (QoL) after hospitalization for COPD exacerbations (6). Though PR for COPD can be personalized to individual patient needs, central elements of PR involve endurance training, strength-building exercises, and breathing techniques (7, 8). These components are coordinated by clinical teams, including nurses, respiratory therapists, physical therapists, and counselors, and, sometimes, by autonomous tools (7). PR for COPD typically consists of three phases, each with ongoing education about how to manage COPD. Phase I (inpatient) occurs while a patient receives care for a COPD-related hospitalization. Phase II (outpatient) is delivered multiple times per week for 6 or more weeks with clinical team oversight. Phase III (maintenance) is an extension of Phase II with less clinical oversight, as patients transition from monitored exercise and treatment to self-management (9).

Enrolling COPD patients in outpatient PR is supported by current evidence, including the majority of observational studies, randomized controlled trials (RCTs), and systematic reviews (10–14). Despite its positive effects, outpatient PR programs remain underutilized, with recent studies showing fewer than 4% of patients hospitalized for COPD participating in PR after discharge (15, 16). Persistent reasons cited by patients who decline PR are a lack of motivation, interest, and perceived benefit (5, 17, 18). The transportation burden associated with PR also drives this lack of adoption, with in-person PR primarily concentrated in urban environments (19). Thus, access disparities are worse in rural areas, with fewer than 12% of rural patients having a PR center within 10 miles from their home (20). Limited insurance reimbursement for PR is an additional challenge for PR programs, exacerbating the already-low participation rates observed in low socioeconomic status (SES) patients (21–23). For those who do enroll in PR, attrition rates are high, often exceeding 50% (24).

To address these barriers, alternative, home-based-PR (HBPR) models that rely on telehealth technologies, have been implemented (25); however, no specific model of HBPR has been defined, with the clinical practice guidelines (2023) from the American Thoracic Society (ATS) on PR in COPD stating only that patients with stable COPD should be offered center- or HBPR options (26). Similarly, a 2021 Cochrane review, including 5 randomized controlled trials (RCTs) and 2 controlled clinical trials (CCTs), compared center-based PR with HBPR, the latter limited to only telephone and videoconferencing modalities (27). Their results found clinically meaningful improvements in exercise capacity, QoL, and dyspnea in both groups and no significant differences in improvements between groups. Notably, participants in HBPR programs were more likely to complete PR than those in center-based programs. Many of these programs offer flexibility beyond that of in-person PR, possibly addressing lifestyle needs and behavior changes critical to improving adherence (28). Moreover, when these programs collect real-time data from wearable devices, they offer clinical teams the opportunity to tailor patient-specific exercise prescriptions and behavioral interventions during outpatient as well as self-management PR phases for sustained impact (6, 29–31).

Several systematic reviews have assessed the effectiveness of wearables for monitoring COPD patients, with none specific to outpatient PR programs. A 2023 meta-analysis (*n* = 37) by Shah et al. showed that wearables, consisting of mostly pedometers, increased mean daily step count and performance on the 6-minute walk distance test over short time periods (30). Moreover, wearables combined with PR programs showed improvements of greater magnitude compared to remote monitoring without coaching or observation, corroborating the results of a 2016 meta-analysis assessing older technologies (32). The use of wearables data to predict or avoid respiratory exacerbations in COPD is indeterminate and had no significant impact on QoL, extending the results of a systematic review of predictive algorithms for at-home COPD monitoring (33). Additionally, a systematic review (included papers, *n* = 7) assessing oxygen saturation and respiratory rate readings from Bluetooth-compatible devices reported high validity in real-world settings, but yielded inconsistent prediction accuracy (34). Additionally, numerous proof-of-concept studies for wearables have only been conducted in controlled environments, like inpatient PR, not accounting for the complexities inherent with real-world deployment (35, 36). No review exclusively focused on HBPR settings has assessed wearable devices. Moreover, the reviews available assess the outpatient and self-management PR phases together, without considering that COPD patients in outpatient PR are normally in a higher-acuity state than those in the self-management PR phase (30, 37).

Despite the promise of objective data from wearable devices, further work is necessary to translate these measures into clinical practice. To our knowledge, no review has surveyed the wearables landscape in HBPR to map the state of currently available digital endpoints: measurable health outcomes from digital technologies used to evaluate whether an intervention has a targeted effect (38). This scoping review primarily aims to identify the set of digital endpoints used to study patient outcomes in HBPR programs for COPD. Second, it aims to summarize gaps in our understanding of using wearable and nearable devices in HBPR to monitor COPD.

## Methods

### Definitions and analytic framework

To organize this scoping review, we conceptualized the HBPR program as monitoring or delivering an intervention to patients with a COPD diagnosis, facilitated by digital sensors embedded in wearable or nearable devices. Included studies were those investigating fully remote outpatient PR implementations. Studies with home health visits and in-person assessments, except in a preliminary phase before HBPR or to provide orientation for HBPR, were excluded. Studies with telehealth visits were eligible for inclusion, provided some digital sensor from a wearable sensor device was a monitoring component of the program.

Eligible studies were those assessing at least one digital endpoint, like change in steps per day from baseline to study completion. Digital endpoints were derived from digital measures, like steps per day, which consist of raw data from sensors in a wearable or nearable device and capture a physical symptom capable of being observed remotely. No specific definitions for devices were applied to limit the scope of emerging technologies included in this analysis. For instance, smartphone app and integrated smartphone app-platform technologies were included if they collected sensor-based data, like smartphone accelerometer data.

We applied this framework to categorize types of digital endpoints and summarize their state-of-the-art implementations in real-world PR programs with COPD patients, thereby highlighting gaps in the literature. Although prior frameworks informed by theory have been proposed to evaluate state-of-the-art technologies for clinical applications, few have focused on assessing digital endpoints. Our approach builds on that of Goldsack et al.'s framework for digital endpoint evaluation. We assembled the set of digital endpoints able to be observed in real-world settings and the devices used to capture them (39, 40).

### Search strategy

This review was conducted according to the PRISMA Extension for Scoping Review guidelines. Searches were conducted across Web of Science core collection [hosted by Clarivate Analytics (≥1,975)], Scopus [hosted by Elsevier (≥1,788)], and Ovid MEDLINE (≥1,946), in addition to Epub ahead of print, in-process, and other nonindexed citations and daily, which is identical to PubMed. Reviewing the literature available at the time of this analysis, studies published from January 1, 2005 through June 1, 2025 were included. Considering the introduction of modern smartphones in the mid-2000s, we selected January 2005 as the starting timepoint for our review. Only publications available in English were included. Observational studies, including both cohort and case-control studies, as well as cross-sectional and mixed-methods studies were eligible for inclusion. Randomized (RCTs) and clinical controlled trials (CCTs) were eligible for inclusion. Reviews, conference abstracts and papers, commentaries/editorials, protocols, and professional society guidelines were excluded. As the scope of this review centered on real-world data capture of symptoms and markers relevant to outpatient PR for COPD, prototype studies as well as those assessing only PR program adherence and usability metrics were excluded. Eligible studies included adults (≥ 18 years of age) with a COPD diagnosis.

**Figure 1:**
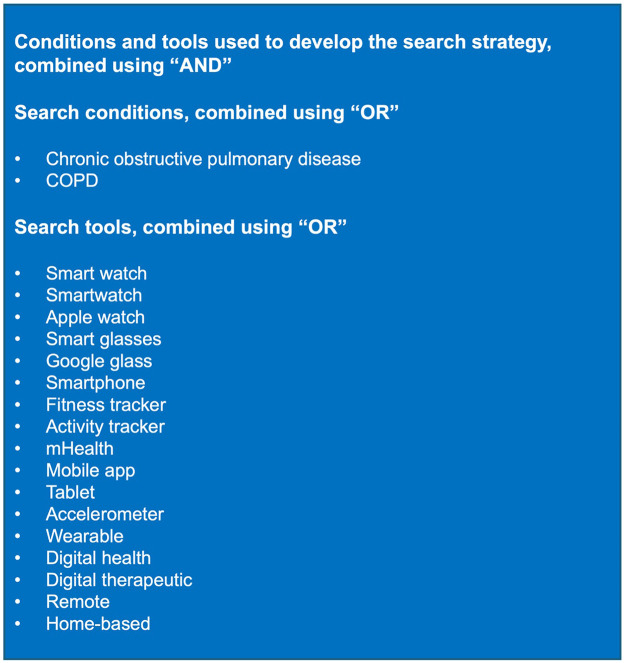
Conditions and tools used to develop the search strategy.

Limiting our search strategy to only studies with “chronic obstructive pulmonary disease” or “COPD” in the title or abstract, a robust set of digital sensor tools were included in the search terms (Figure 1).

### Selecting studies

First, title and abstract screening for relevance was performed by one investigator (SJZ). At this step, the list of studies was reviewed by 3 additional investigators (CAC, LJF, and EF). Second, a full-text review, including annotation and adjudication, was completed by two investigators (SJZ and CAC). The final list of included studies was reviewed by a clinical expert to ensure relevance (RJB). The rationale for each eligibility criteria applied is available in Table 1.

**Table 1:** Eligibility criteria and rationale for study inclusion and exclusion.

Eligibility criteria and variable	Rationale
Inclusion criteria	
Outpatient PR phase	Participants must be participating in an outpatient PR program, with data collection at the beginning, end, or during the program
Fully remote setting	Data must be collected outside of clinical environments and in real-world settings
Participants exclusively with a COPD diagnosis in the observational or experimental cohort	Participants must be diagnosed with COPD, as evidenced by clinical documentation or assessment
Peer-reviewed research	Only studies published in peer-review journals will be included, due to their credibility
Wearable or nearable device	Tools used to collect digital endpoint data must be from a wearable device, like a smartwatch, or a nearable device, like a metered inhaler
Empirical study design with observational, comparative, and/or clinical trial components	Studies must address a hypothesis or research question
Studies published from January 1, 2025 through June 1, 2025	Included studies conducted over the past two decades to provide a comprehensive analysis of all digital endpoints proposed
Articles published in English	Limited due to language proficiency of investigators
Participant sample data included	Articles must include the number of participants, at minimum
Exclusion criteria	
Studies including participants without COPD diagnosis	Aside from healthy controls, studies must include participants with COPD diagnoses to minimize disease-related biases
Nonempirical studies	Editorials, guidelines, reviews, brief reports, and other articles without a clear hypothesis or research question were excluded to minimize viewpoint bias
Studies focused exclusively on feasibility, usability, acceptability, or adherence	Though pilot and feasibility studies with explicit research questions involving digital endpoints were eligible for inclusion, studies exclusively focused on proof-of-concept or qualitative work were excluded from this review of sensor-generated digital endpoints
Studies focused on inpatient (Phase 1) or self-management (Phase 3) phases of PR	Studies focusing on phase 1 or 3 of PR were excluded
Device or algorithm validation studies	Considering this review aimed to map the state-of-the-art in digital endpoints for remote PR in COPD patients, studies proposing new devices or predictive algorithms were not appropriate for inclusion
Wearable or nearable device implementation with no sensor-based data analyzed	Given that this review focused on digital endpoints, any studies where wearable and nearable devices were used during PR but their resulting data not analyzed were excluded
Studies focused exclusively on qualitative data, including patient-reported surveys submitted via nearable and wearable devices	Studies with only qualitative data or only patient-reported surveys, such as those submitted via smartphone apps, were excluded, as they are not sensor-based endpoints

### Data collection and analysis

The following data was extracted by one investigator (SJZ): authors, year, country, study design, COPD severity, sample characteristics, wearable/nearable device, digital endpoints, results, and attrition rate. When reported in studies, non-digital endpoints, like patient-generated surveys or in-person walking tests, were not extracted for evaluation. Similarly, when a wearable or nearable device was used in a study but no recorded data were analyzed, the study was excluded. Digital measures used in descriptive studies were excluded from this analysis of digital endpoints. Smartphones were only listed as a monitoring device used to generate digital endpoints if they enabled the collection of sensor-based data, regardless of the smartphone's role in the study.

## Results

**Figure 2:**
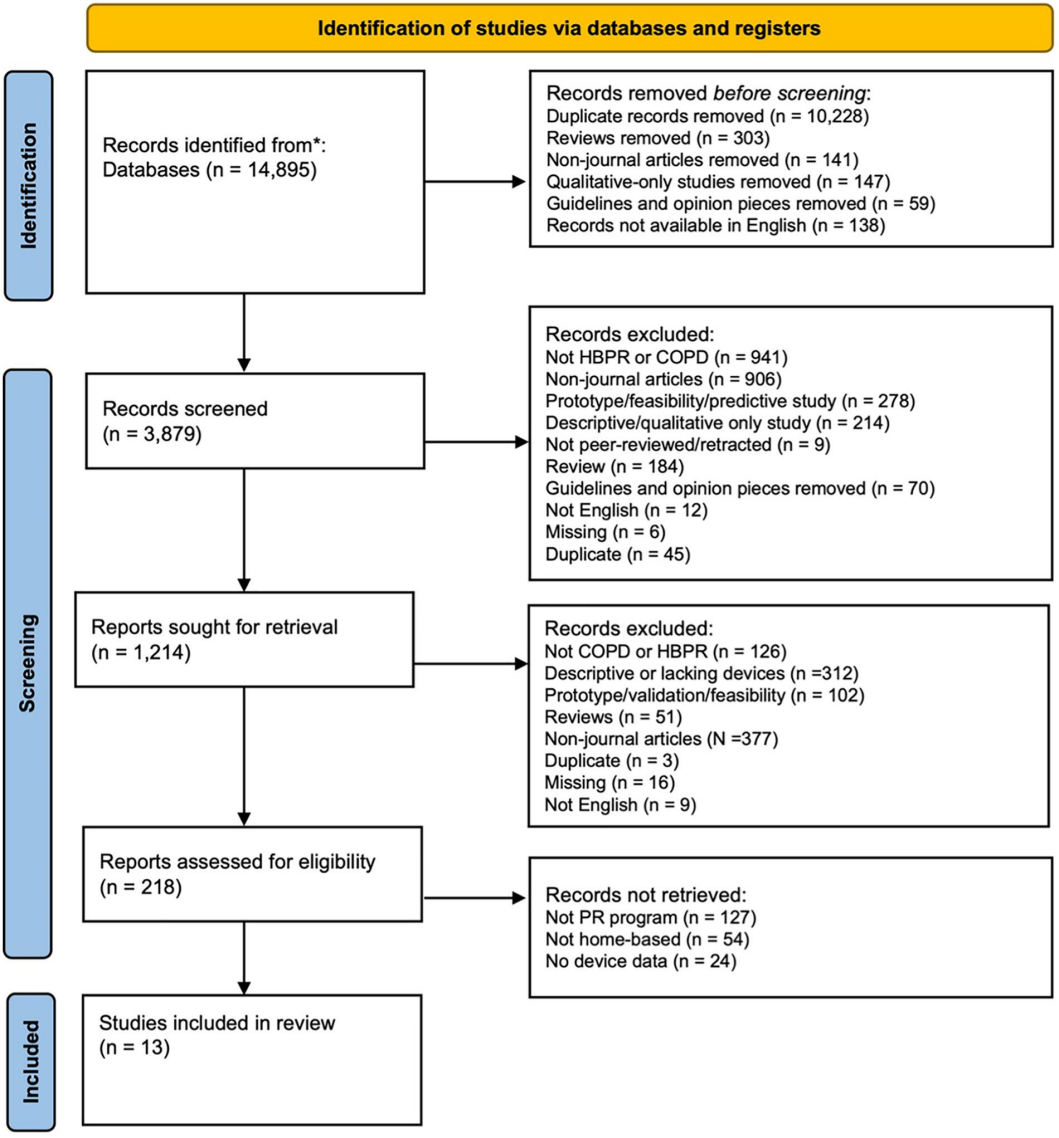
PRISMA flowchart of included studies.

This initial search generated 14,895 articles. After removing duplicate articles, and then screening titles and abstracts, 218 titles were selected for full-text review. Of the 218 articles adjudicated, 6.0% (*n* = 13) met the inclusion criteria. The PRISMA flow chart for this search is detailed in Figure 2.

### Study characteristics

Summary data for each study is reported in Table 2. Most studies (*n* = 8, 61.5%) were published after 2019. For study design, 10 articles reported a randomized controlled trial (RCT), 2 used an observational cohort, and 1 was a secondary analysis of an RCT. More than half of the studies (69.2%) enrolled cohorts of fewer than 100 COPD patients. The average duration of PR for studies was 12.5 weeks (min = 4 weeks; max = 52 weeks). One study had a 6-month follow-up checkpoint, and another had a 12-month checkpoint. All studies focused on adult populations, primarily consisting of middle-aged and older adults, with the youngest cohort mean age of 64 years. Three studies lacked participant age and sex data (46–48). Of the studies with participant demographic data, half enrolled primarily male participants. Only 2 studies (42, 45) included race and ethnicity data of participants.

**Table 2:** Summary of studies on digital endpoints in HBPR for COPD.

Author, Year	Country	Study Design; Devices	COPD Severity	Sampling	Sample Characteristics—Experimental	Sample Characteristics—Control	Digital Measure (w/Sample Frequency)	Digital Endpoint	Results	Attrition Rate
Benzo et al. (41)	Sweden	8-week Prospective Cohort; wrist-worn activity tracker (Garm*in vivo*fit 4) worn throughout HBPR	COPD diagnosis	Recruited from hospitals, health care units, websites, and lung facilities (Stockholm and Sweden)	*N* = 12; 33.3% male; mean age (SD) = 71 (55–83); Race unknown; Ethnicity unknown	N/A	Steps/day	Change in steps/day from baseline to week 8	No significant change in monthly steps from baseline to end. Minimal clinical improvement (500 steps/day) found in 8 participants.	8%
Benzo et al. (42)	US	12-week RCT; wrist-worn activity tracker (Garm*in vivo*fit 4) worn throughout PR	Diagnosis of COPD confirmed by records	Recruited (Mayo Clinic MN and FL and Health Partners, Minneapolis–Saint Paul, MN)	*N* = 188; 46.3% male; mean age (SD) = 69 (10); 94.1% White; 1.1% Hispanic	*N* = 187; 40.6% male; mean age (SD) = 69 (10); 96.3% White; 0.5% Hispanic	Daily: total sleep time (min), steps, LPA (min), MVPA (min)	Change in total sleep time/day, steps/day, LPA/day, and MVPA/day from baseline to week 12	HBPR: sleep, steps, LPA, and MVPA significantly improved by end of PR.	24%
Cerdan-de-las-heras et al. (43)	Denmark	8-week prospective Cohort; wrist-worn accelerometer (ActiGraph Monitor wGT3X-BT) worn for 7 days at baseline and after 8 weeks, 3 months, and 6 months	Physician-verified COPD	Recruited from outpatient clinic (Department of Respiratory Diseases and Allergy, Aarhus University Hospital)	*N* = 27; 51.6% male; mean age (SD) = 67 (10); Race unknown; Ethnicity unknown	*N* = 27; 48.4% male; mean age (SD) = 73 (7); Race unknown; Ethnicity unknown	Steps/week; total vector magnitude counts per minute (VMCPM)	Change in steps/week and VMCPM from baseline to week 8	No significant change in weekly steps of VMCPM from baseline to end.	57%
Chaplin et al. (44)	UK	7-week RCT; chest-worn accelerometer (SenseWear Armband) worn 7 days before and after PR	COPD diagnosis (postbronchodilator FEV_1_ of <80% and FEV_1_ forced vital capacity ratio of ≤0.70), GOLD stage 2–4	Unknown	*N* = 20; 90.0% male; mean age (SD) = 68 (7); Race unknown; Ethnicity unknown	*N* = 19; 55.9% male; mean age (SD) = 67 (9); Race unknown; Ethnicity unknown	Steps/day, mean bouts of moderate activity for ≥2, ≥5, ≥10, and ≥20 min	Change in steps/day and mean bouts of moderate activity for ≥2, ≥5, ≥10, and ≥20 min from baseline to week 7	No significant change in daily steps of moderate activity from baseline to end.	48%
Federman et al. (45)	US	6-month RCT; actuation sensor-enabled inhaler (Doser Electronic Monitor or SmartDisk) used daily for 6 months	Chart-documented severe/very severe COPD (FEV_1_ < 50%)	Recruited from outpatient general medicine and pulmonary practices (Mount Sinai Hospital in East Harlem)	*N* = 30; 36.7% male; mean age (SD) = 64 (8); 6.7% White; 56.7% Black; 36.6% Hispanic	*N* = 29; 37.9% male; mean age (SD) = 64 (8); 10.4% White; 37.9% Black; 51.7% Hispanic	Inhaler use/day	Change in inhaler use/day from baseline to month 6	Daily inhaler use increased (difference-in-differences 7.7%, 95% CI = −29.6%–45.0%)	14%
Godtfredson et al. (46)	Denmark	12-month RCT; thigh-worn accelerometer (ActivPAL triaxial accelerometer) worn 5 days before and after PR	Severe COPD	Recruited from 8 PR sites (Capital Region of Denmark)	*N* = 45; unknown % male; mean age unknown; Race unknown; Ethnicity unknown	*N* = 39; unknown % male; mean age unknown; Race unknown; Ethnicity unknown	Steps/day, sedentary behavior (min), active behavior (min)	Change in steps/day, sedentary behavior/day and active behavior/day from baseline to month 12	HBPR: No significant changes were observed; Control: Steps/day significantly decreased in the control group [−822 steps/day; 95% CI = (−1,575, −138)].	37%
Hansen et al. (47)	Denmark	10-week RCT with 22-week follow-up; thigh-worn accelerometer (ActivPAL triaxial accelerometer) worn 5 days before and after PR	COPD diagnosis (FEV1/FVC <0.70, FEV1 < 50%)	Recruited from 8 university hospitals (Greater Copenhagen)	*N* = 67; unknown % male; mean age unknown; Race unknown; Ethnicity unknown	*N* = 67; unknown % male; mean age unknown; Race unknown; Ethnicity unknown	Steps/day, sedentary behavior (min), active behavior (min)	Change in steps/day, sedentary behavior/day and active behavior/day from baseline to week 10	HBPR: steps/day were unchanged from the start to end. Control: steps/day significantly decreased from baseline to the end of the intervention and, again, to the follow-up period (22 weeks).	0%
Horton et al. (48)	UK	Secondary Analysis of 7-week RCT with 6-month follow-up; armband activity tracker (SenseWear Armband) worn 5 days before baseline, after 7 weeks, and after 6 months	Not discussed	Recruited participants referred to PR from Glenfield Hospital (Leicester, UK)	*N* = 26; unknown % male; mean age unknown; Race unknown; Ethnicity unknown	*N* = 25; unknown % male; mean age unknown; Race unknown; Ethnicity unknown	Steps/day, bouts of moderate PA [3–6 metabolic equivalent of tasks (METs)] for > 10 min (min), sedentary behavior (<2 METs) (min)	Change in steps/day, bouts of moderate PA for > 10 min, and sedentary behavior from baseline to week 7	HBPR: steps/day significantly increased from baseline to end, compared to the center-based PR cohort (mean difference 62 min: 95% CI −56 to 248, *p* = 0.24). Time spent sedentary was also decreased in the HBPR cohort (CI −106 to 2; *p* = 0.039). No significant difference was found for time spent in MPA. At 6 months, step count and time spent in MPA for both cohorts reverted to baseline.	67%
Paneroni et al. (49)	Italy	40-day RCT; pedometer (Omron Walking Style II, HJ−720IT pedometer) worn 3 days before and after PR	COPD diagnosis (FEV1 < 50%)	Recruited participants referred to the Salvatore Maugeri Foundation Institute of Lumezzane (Brescia)	*N* = 18; 89% male; mean age (SD) = 66 (11); Race unknown; Ethnicity unknown	*N* = 18; 83% male; mean age (SD) = 66 (6); Race unknown; Ethnicity unknown	Steps/day	Change in steps/day from baseline to day 40	HBPR: significantly increased physical activity (3,412 vs. 1,863 steps/day, *p* = 0.0002).	0%
Tabak et al. (50)	Netherlands	4-week RCT; belt-worn accelerometer sensor + smartphone (3D-accelerometer MTx-W sensor) used at least 4 days/week for 4 weeks	COPD diagnosis	Recruited from pulmonary medicine at Medisch Spectrum Twente hospital (Enschede)	*N* = 15; 60.0% male; mean age (SD) = 66 (9); Race unknown; Ethnicity unknown	N/A	Steps/day	Change in step/day from baseline to week 4	After discouraging cue: steps/min significantly decreased in the 30 min after (*p* < 0.001); After encouraging cue: significantly increased in the 10 min after < *p* < 0.05). Compared to baseline, steps/min increased by 13% (*p* = 0.008) during the intervention.	unknown
Tabak et al. (51)	Netherlands	4-week RCT; belt-worn accelerometer sensor + smartphone (3D-accelerometer MTx-W sensor) used at least 4 days/week for 4 weeks	COPD diagnosis	Recruited by physician or nurse practitioner	*N* = 14; 57.1% male; mean age (SD) = 65 (1); Race unknown; Ethnicity unknown	*N* = 16; 68.8% male; mean age (SD) = 68 (6); Race unknown; Ethnicity unknown	Steps/min	Change in steps/min from baseline to week 4	No significant change in daily steps from baseline to end.	12%
Tsai et al. (52)	Austrailia	8-week RCT; armband activity tracker (SenseWear Armband) worn for 6 days before and after PR	Stable COPD diagnosis (FEV1/FVC <70% and FEV1 < 80% predicted post-bronchodilator)	Recruited participants referred to a tertiary hospital PR programme (Sydney)	*N* = 19; 63.2% male; mean age (SD) = 73 (8); Race unknown; Ethnicity unknown	*N* = 17; 35.3% male; mean age (SD) = 75 (9); Race unknown; Ethnicity unknown	Daily: total energy expenditure (>3 METs), steps, metabolic equivalents (METs)	Change in total energy expenditure/day, steps/day, and METs/day from baseline to week 8	No significant change in daily steps or energy expenditure from baseline to end.	3%
Vasilopoulou et al. (53)	Greece	2-month RCT with 12-month follow-up; wrist-worn accelerometer (ActiGraph Monitor wGT3X) worn throughout PR	Stable COPD diagnosis (FEV1/FVC <70% and FEV1 < 80% predicted post-bronchodilator)	Recruited participants attending Outpatient Clinic at 1st Dept of Respiratory Medicine at Athens University Medical School (Sotiria General Chest Hospital, Athens, Greece)	*N* = 47; 93.6% male; mean age (SD) = 67 (10); Race unknown; Ethnicity unknown	Hospital-based PR *N* = 50; 76.0% male; mean age (SD) = 67 (7); Usual care *N* = 50; 74.0% male; mean age (SD) = 64 (8); Race unknown; Ethnicity unknown	Daily: steps, LPA (min), moderate PA (min), lifestyle PA (min), sedentary (min)	Change in steps/day, LPA/day, moderate PA/day, lifestyle PA/day, and sedentary/day from baseline (end of RCT) to month 12	HBPR: significant decrease in sedentary behavior and increases in light, lifestyle, and moderate PA from baseline to end, compared to center-based PR. These changes were more pronounced compared to usual care without PR.	2%

### What are the most frequently used digital endpoints in HBPR for COPD?

**Figure 3:**
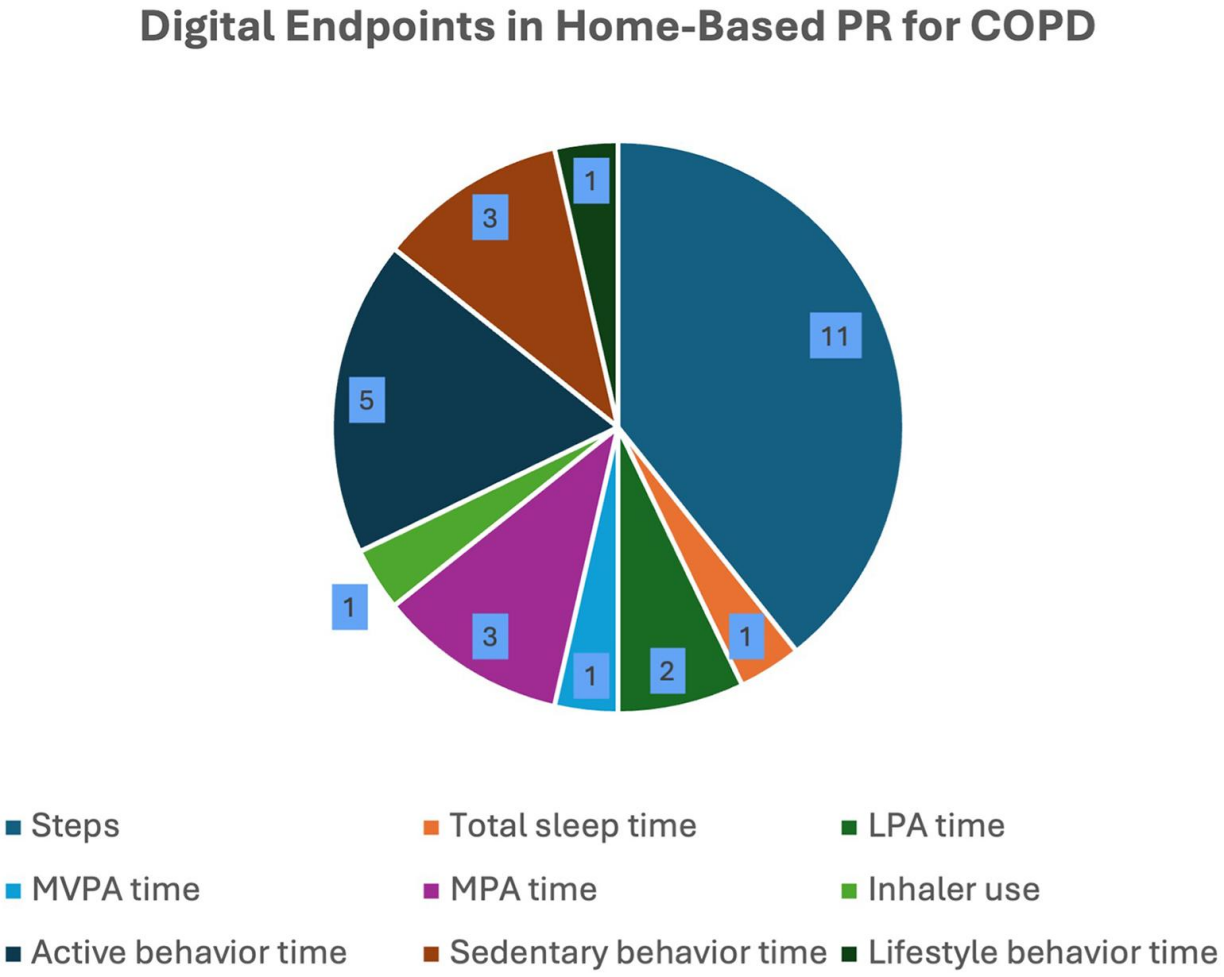
Frequency of digital endpoints used.

Among the articles included, all endpoints were generated using daily summaries of wearable data. Five studies (38.5%) examined more than 1 digital endpoint listed in Figure 3 (42, 45–47, 52, 53). Step count was the most common endpoint examined (84.6%), with most studies assessing steps/day and only 1 tracking steps/min. The next most popular endpoints were minutes spent in active behavior (38.5%), minutes spent in sedentary behavior (30.8%), minutes spent in moderate physical activity (23.1%), minutes spent in light physical activity (15.4%), minutes spent in total sleep (7.7%), minutes spent in moderate-to-vigorous physical activity (MVPA) (7.7%), minutes spent in lifestyle behavior (7.7%), and inhaler use (7.7%). One implementation of the “minutes spent in active behavior” endpoint was assessed within the 30 min of a motivational cue (intervention) delivered to patients via smartphone (51).

### What are the most frequent devices used to measure digital endpoints in COPD?

**Figure 4:**
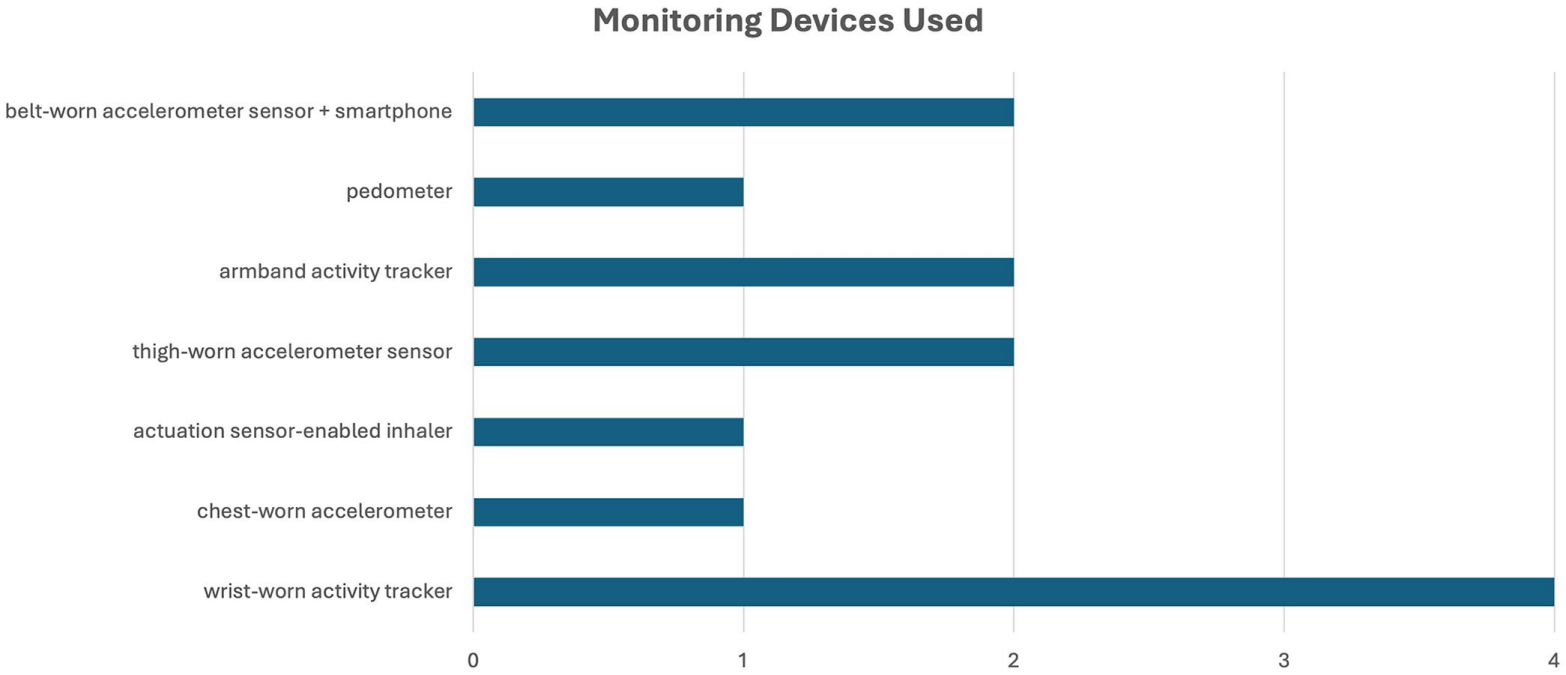
Frequency of monitoring devices used.

To generate digital endpoints, the most frequently used monitoring strategy employed a wrist-worn activity tracker (*n* = 4), followed by an armband activity tracker (*n* = 2), thigh-worn accelerometer sensor (*n* = 2), and belt-worn accelerometer sensor + smartphone (*n* = 2) (Figure 4). Eight studies used only wearables, and 4 studies used a smartphone plus nearable approach. One study used only a nearable (actuation sensor-enabled inhaler). Detailed information about the devices used to measure digital endpoints in the included studies is available in Supplementary Table S1.

### How are digital endpoints used?

Of the 13 studies included, all captured sensor-based data from participants to assess a HBPR program.

Ten studies were RCTs, each with unique HBPR programs (42, 44–47, 49–53). Hansen et al. (2020) compared the outcomes of two supervised HBPR programs using a tri-axial accelerometer taped to a participant's thigh: one at home (*n* = 67) and another conducted in clinical settings (*n* = 67) (47). From baseline to the end of the 22-week follow-up, a statistically significant decrease in steps/day was observed in the conventional PR group (*p* < 0.05); however, no change was observed in the HBPR group. Using the same accelerometer, Godtfredson et al. (2020) examined physical activity level (PAL) differences between HBPR (*n* = 45) and standard PR (*n* = 39), using three measures: sedentary time, active time, and steps/day (46). Twelve months after PR, no significant differences were observed for PAL in the HBPR group but steps/day decreased in the control group [−822 steps/day; 95% CI = (−1,575, −138)]. Extending these results, Benzo et al. (42) used a wrist-worn activity monitor, demonstrating that a fully remote, unsupervised HBPR program (*n* = 143) could deliver a significant improvement in steps/day by the end of 12 weeks (*p* = 0.0113) compared to standard PR (*n* = 142); however, no clinically meaningful improvements were noted for time spent in moderate-vigorous physical activity (MVPA), light PA, or sleep (42). Chaplin et al. (44) used a chest-worn accelerometer to compare a conventional PR cohort (*n* = 51) with a web-enhanced HBPR cohort with enhanced exercise and educational training (*n* = 52) (44). While no significant findings were observed in the accelerometer data collected before and after the PR interventions, a nonsignificant increase (12%) in steps/day was observed in the web-based PR cohort (5,645–6,112 steps/day), primarily consisting of short MVPA episodes.

One study assessed medication use in a cohort of participants offered HBPR (*n* = 25), with 75% participating (45). Federman et al. (45) used electronic monitoring inhaler devices to quantify medication use remotely in a digital symptom-management platform study, finding that participants in the HBPR program, who were instructed to use their inhaler on a daily basis, exhibited increased daily medication use (difference-in-differences = 7.7%) compared to controls without PR (*n* = 26) (45).

Two studies used a platform strategy for monitoring, which consisted of a Bluetooth-connected belt-worn accelerometer sensor that paired with a participant's smartphone, also worn on the belt for the study (50, 51). In the first, Tabak et al. (50) compared a HBPR program (*n* = 14) to standard care (*n* = 16), finding that pedometer-measured steps/day did not improve (51). In the second, Tabak et al. (50) deployed an autonomous coaching program, that aimed to adjust PA behaviors in real-time via text messaging and visuals, like graphs (50). Participants (*n* = 15) responded with appropriate modified behaviors, decreasing PA after a discouraging cue and increasing PA after an encouraging one (*p* < 0.05). In addition, overall activity levels increased in the cohort; however, although the intervention was designed to more evenly distribute PA across daytime hours, no balance in PA was achieved.

Using a wrist-worn accelerometer, Vasilopoulou et al. (53) investigated the effectiveness of a maintenance HBPR program, one that participants could complete after a primary PR program, if necessary (*n* = 147) (53). Considering positive effects with the initial PR program—namely, increased light, lifestyle, and moderate activities and decreased sedentary time (*p* < 0.05), sustained improvement in all 4 physical activity metrics was observed after the 12-month HBPR program.

Tsai et al's (52) study began with an at-home visit to help prepare participants for the HBPR program (52). This study compared HBPR (*n* = 19) to a control group (*n* = 17) that received standard care without exercise training, no significant between-group differences in SenseWear armband-measured steps/day were observed at the conclusion of the HBPR. Also using the armband, Horton et al. (48) launched a study with an in-hospital preliminary component to orient participants to the HBPR program (48). They compared the results of a HBPR program (*n* = 26) to that of standard, center-based PR (*n* = 25). Although the HBPR cohort showed promising improvements at the end of 7 weeks, with a significant increase in step count (*p* = 0.006) and decrease in time spent sedentary compared to the control group (*p* = 0.039), the step count for both groups reverted to baseline after 6 months. Though no statistically significant change in moderate physical activity (MPA) was observed during the study, time spent in MPA also returned to baseline for both cohorts by the 6-month mark. In contrast, Paneroni et al. (49) used a pedometer to compare the impact of HBPR (*n* = 18) vs. standard PR (*n* = 18), finding increased physical activity in HBPR participants at study conclusion (3,412 v. 1,863 steps/day; *p* < 0.001) (49). Cerdan-de-las-heras et al. (43) applied a wrist-worn accelerometer to track 7-day steps/day and total vector magnitude counts per minute (VMCPM) (43). No difference from study commencement to end or between HBPR (*n* = 27) or control cohorts (*n* = 27) was observed for either digital endpoint.

Benzo et al.'s (41) small-scale evaluation (*n* = 12) of a remote coaching program found an overall average increase in monthly step counts, but no statistically significant results derived from monitoring devices; however, individual participant metrics improved over the 8-week study, with 8 participants logging 500 daily step counts (41).

## Discussion

To our knowledge, this is the first scoping review to map digital endpoints used in HBPR for COPD studies. In addition, our objective was to identify the devices most frequently used to capture data for these digital endpoints. Most were published after 2020 and enrolled fewer than 100 COPD patients. While the most common endpoints derived from objective data were daily step count and daily minutes spent in active behavior, multiple studies assessed active behavior by different intensity levels, from lifestyle activities like walking to MVPA. Activity trackers were the most common devices used to capture data, and RCTs were the most commonly used study design. As studies recorded conflicting outcomes, with one showing HBPR participants returning to their lower, baseline PA levels after follow-up periods, it remains to be seen whether self-management programs, implemented after the outpatient PR phase, can sustain the benefits of HBPR for participants. For the studies using similar digital endpoints, conflicting results might be explained by HBPR intervention design or patient characteristics, to name a few reasons. Alternatively, conflicting results might be modulated by endpoint reliability, an output of wearable device usability. Devices used in HBPR programs vary, as researchers and service design teams select tools based on site-specific factors, including state regulations for remote monitoring devices (54). The heterogeneity of study designs and devices used to measure endpoints limits comparative analyses and meta-analyses in this research area; however, if HBPR programs are ever to be sustainable in the long-run, methodological guidance is necessary regarding the use of wearables.

Most studies included endpoints capturing physical activity. Given that exercise capacity is an indirect measure of lung function and COPD status, it is not a surprise that most studies evaluated whether an intervention could improve physical activity levels (55). Increased PA is a key measure for successful completion of PR programs, one that is easy to objectively measure in real-world settings with high-quality consumer wearables (56). Across studies comparing steps per day and other physical activity metrics, conflicting results regarding the efficacy of HBPR were found. Given that little is known about participant profiles of those who would benefit most from HBPR, targeted recruitment of studies in COPD monitoring might be necessary to standardize digital endpoints for more efficient interpretation (57). Though it is important to consider variations in results may be driven by different levels of COPD severity that were not controlled for in analyses, the need for clarity here is critical to inform future studies.

The trend of converging studies around physical activity monitoring is evident. Though valuable for investigating specific targets when evaluating PR programs, this approach disregards the complexity of factors, from environmental to lifestyle behaviors, contributing to HBPR outcomes, as well as COPD exacerbations and recovery (31, 58). Considering the routine practice of charting lung function at baseline and follow-up for PR program participants in clinical or laboratory environments, it is surprising that no endpoint used a patient-facing spirometer for home-based assessment of lung function. Only the actuation sensor-enabled inhaler was interpreted as an indirect measure of lung capacity, with participants instructed to use their inhaler daily and the endpoint quantifying compliance with daily use guidance. It is possible that some researchers are unaware of modern smart inhaler and spirometer devices or that the reliability of remote spirometer devices has yet to be validated in COPD populations. Regardless, as respiratory disease prevalence grows over the next decade, nearables like smart breathalyzers could fill this need in HBPR programs (59). Compared to wearable devices, the use of nearable devices in clinical remote monitoring is in its infancy and is reflected in the finding that only one nearable device study met the inclusion criteria for this review.

Similarly, as COPD patients frequently report sleep disturbances, it is remarkable that only 1 study assessed a digital endpoint for sleep (total sleep time) (60). With an ever-growing number of digital sleep interventions, like digital cognitive behavioral therapy, and digital sleep measures, like nighttime respiratory rate, tracking sleep in COPD programs is ripe for discovery (61). Also, no digital endpoints evaluated cardiovascular markers, which frequently predict short- and long-term prognosis in COPD (62).

Most studies that met the inclusion criteria paired multiple devices. Despite the variety in devices used, not all data was translated into digital endpoints. For instance, Vasilopoulou et al. used a Bluetooth-enabled pulse oximeter, wearable tracker, and novel smartphone app (53); however, no data from either the pulse oximeter or wearable tracker was converted into a study endpoint. This highlights the gap between available technologies, from Bluetooth-linked inhalers to heart rate-capturing armbands, and clinically meaningful interpretations of the data they generate. Compared to real-world digital endpoints in fields like cardiology, which can involve heart rate and blood pressure endpoints, outcomes evaluated by digital endpoints for HBPR monitoring were primarily restricted to pedometer-derived step counts and accelerometer-derived physical activity (63). Considering the ever-growing set of digital measures derived from high-quality devices, like nighttime heart rate variability, or emerging digital measures calculated by machine learning techniques, like smartphone audio sensor-derived cough detection, the potential for growth in the HBPR digital endpoint space is high (64, 65).

Additionally, the results of Benzo et al's study, which found no significant results between cohorts, but found improved step counts for individuals over their observational period, support the need for evaluative methods that incorporate patient-specific thresholds in lieu of population reference thresholds (41). Although using digital endpoints for HBPR monitoring appears feasible, no recommendations regarding optimal tool selection, standardized methods, or sampling frequencies for sensor-based data have been established. Despite daily physical activity being the strongest predictor of survival in COPD patients, the measure is underutilized (66). The lack of validated or standardized physical activity measures specific to COPD patient populations—and the need for different digital endpoints to help clinicians assess whether individual patients have engaged in distinct types of physical activity, from walking to weightlifting and everything in between, remains a key barrier to evaluating HBPR.

## Limitations

The main limitation is the scope of this review, which necessitated broad inclusion criteria rather than focusing on specific device types or on endpoints. With multiple included RCTs enrolling fewer than 100 participants, the findings from this review may be biased against observational real-world data. As this was a scoping review, we could not conclude which digital endpoints were valid to assess HBPR; however, this highlights a gap in the literature for future work.

Considering that we limited our study to digital endpoints, this review reflects only the current state of digital endpoints and not the totality of potential endpoints that may be in the early stages of development, like audio-based exacerbation measures or EEG headbands to measure sleep (40, 64). Third, our search criteria excluded studies of patients at risk of or with suspected COPD, focusing exclusively on patients with a COPD diagnosis. Thus, other digital endpoints relevant to PR monitoring may exist but may have only been evaluated in the pre-diagnostic phases. We also focused on HBPR, rather than home-based self-management, and may have excluded digital endpoints that are used in the latter category. While other digital endpoints relevant to PR monitoring may exist in prediagnostic phases for patients at risk of or with suspected COPD, or for home-based self-management phases, the goal of this study was to focus on HBPR due to the existing gap in knowledge regarding this phase, as COPD patients in this phase are typically in a high-acuity state.

In this scoping review, we conducted neither a risk-of-bias assessment nor quality analysis of included HBPR articles, both of which remain uninvestigated in peer-reviewed literature. Although we included data privacy and security information for devices included in this study (Supplementary Table S1), no discussion of the cost-effectiveness of wearables used in HBPR vs. tools used in hybrid implementations, such as when patients return to clinical settings at specific timepoints to perform routine physical assessments, was included (21).

Another key limitation inherent in PR programs is that patients with stage 4 COPD are less likely to be referred to PR, whether center- or home-based, potentially restricting the generalizability of these findings to patients with less severe COPD (66). As the season of monitoring in PR is known to impact participant activity levels but was not reported in studies included in this review, this should also be considered when interpreting these results (67). Lack of knowledge about wearable and nearable usability and acceptability in COPD patients participating in HBPR is a barrier to developing digital endpoints. Devices and platforms designed with user-centric methods, accounting for the high comorbid burden and functional impairments characteristic of patients in HBPR, could improve adherence and generate more reliable data to assess HBPR outcomes (68). To identify the most meaningful digital endpoints and thresholds for HBPR programs, factors influencing patient adherence to using wearable and nearable devices must be better understood. Another barrier to the development of digital endpoints is that psychosocial aspects of device use in HBPR are understudied in COPD patients. For instance, some HBPR patients may want to read automated reports of their daily progress while others might want to receive information verbally from clinicians. Others might benefit from training with wearable devices before HBPR participation. With little information about the sociodemographic status of participants in HBPR, as only 2 of the 13 included studies reported race and ethnicity data, it is plausible that culturally tailored device designs are requisite for the development of digital endpoints that do not exclude patients from underserved groups.

Lastly, limited peer-reviewed information is available regarding privacy and security specific to the devices included in this study. The wearable sensor paired with smartphone application is no longer supported by its manufacturer, potentially exposing study participants to vulnerabilities (51). Only the wearable pedometer manufacturer explicitly mentioned that it does not collect geographic location data (49). Garmin and ActiGraph both disclosed ongoing compliance with best privacy and security practices. More research into privacy and security concerns of COPD patients and clinicians overseeing HBPR is needed to address barriers to data collection in HBPR.

## Future directions

This study has important clinical research implications. To date, caution must be exercised when interpreting remotely collected HBPR data and clinicians should engage HBPR patients via conventional monitoring means, such as video telehealth and in-person visits, to ensure accurate digital endpoint data derived from wearable devices. It remains unclear what roles individual wearable device usability and acceptability play in generating data for digital endpoints in HBPR programs.

This scoping review highlights the following areas for future investigation. First, considering the heterogeneity of randomized controlled trials and observational studies with digital endpoints in HBPR programs, little is known about optimal COPD patient sampling frequencies, or which populations would benefit most from integrating specific wearables in HBPR. As such, future research should investigate factors influencing wearable device use in HBPR as well as the impact of study design characteristics, like variation in program duration, on adherence. Second, researchers should prioritize recruiting larger sample sizes to enhance the generalizability of findings and validate wearables for capturing digital endpoint data in the COPD population. Through large-scale validation studies, thresholds that capture clinically meaningful changes in digital measures can be established and robust systematic reviews performed.

With the growing number of multi-sensor platforms, such as those with wearables in addition to nearable ambient sensors, AI-derived digital endpoints, such as the classification of coughing sounds from microphone data, will potentially augment current digital endpoints and require additional validation in COPD cohorts. Nearable-wearable integration platforms might also help to augment current clinical profiles of COPD severity as well as discover COPD phenotypes. Recording objective measures of COPD severity, like FEV1, in future HBPR studies could inform the development of more precise digital endpoints stratified by COPD severity.

## Conclusions

This scoping review revealed that (1) digital endpoints in HBPR primarily focus on physical activity, (2) digital endpoints tracking sleep, spirometer values, and other cardiovascular biomarkers are understudied, (3) some data streams from wearable devices have yet to be translated into endpoints for HBPR, and (4) optimal sampling frequency for the digital endpoints in this space is unclear. Future investigations should prioritize robust inclusion criteria for HBPR, to enhance the standardization of digital endpoints by recruiting participants for HBPR who are most likely to benefit, and explore relevant sleep and cardiovascular measures, like nighttime respiratory rate and heart rate, as digital endpoints for HBPR evaluation (69–72).
