# Supplementary material for: Using wearable and nearable devices in telerehabilitation for COPD: a scoping review of digital endpoints in home-based programs
Source: Front Digit Health. 2026 Feb 3;8:1698019. doi: 10.3389/fdgth.2026.1698019 (PMC12909527; doi:10.3389/fdgth.2026.1698019)
Supplement: Supplementary file 1 [file Table1.docx]

|  | Validation in Chronic Disease Population? | Validation in Respiratory Disease Population? | Reliability of Results | Cybersecurity Landscape |
| --- | --- | --- | --- | --- |
| Garmin Vivofit 4 | No | No | Little evidence supports the validity of the commercially-available Garmin Vivofit 4 to measure step counts in COPD patients in real-world settings. One validation study in real-world settings with older adults found that the wearable underestimates steps at slow speeds and in indoor settings.  Reference: https://journals.humankinetics.com/view/journals/japa/30/5/article-p833.xml | Garmin guarantees 24/7 threat monitoring and routine security patches and updates. Garmin also offers a security issue report process. No peer-reviewed literature is available on Garmin Vivofit 4 cybersecurity specific to clinical monitoring. |
| ActiGraph Monitor wGT3X-BT | Yes | No | Growing evidence supports the validity of the research-grade ActiGraph Monitor wGT3X-BT to measure physical activity energy expenditure in real-world settings. One validation study found that the wearable underestimates physical activity energy expenditure in patients with cardiovascular risk factors.  Reference: https://journals.plos.org/plosone/article?id=10.1371/journal.pone.0309481 | ActiGraph complies with applicable General Data Privacy Regulation (GDPR). ActiGraph uses Amazon and Microsoft vendors for data storage. No peer-reviewed literature is available on ActiGraph Monitor wGT3X-BT cybersecurity specific to clinical monitoring. |
| SenseWear Armband | No | No | Little evidence supports the validity of the research-grade SenseWear Armband to measure step counts in COPD patients in real-world settings. One validation study with an older device model in laboratory settings with healthy adults found that the wearable is less precise than gold-standard indirect calorimetry.  Reference: https://pmc.ncbi.nlm.nih.gov/articles/PMC5360603/#abstract1 | No peer-reviewed literature is available on SenseWear Armband cybersecurity specific to clinical monitoring. |
| Doser Electronic Monitor or SmartDisk | Yes | Yes | The results of one validation study suggest that the passive data on inhaler use obtained via the nearable sensor(s) are more accurate than patient-reported measures.  Reference:  https://www.tandfonline.com/doi/full/10.1080/15412555.2020.1712688#abstract | No peer-reviewed literature is available on Meditrack Doser Electronic Monitor or Advair SmartDisk cybersecurity specific to clinical monitoring. |
| ActivPAL (triaxial accelerometer) | Yes | No | Growing evidence supports the validity of the research-grade ActivPal to measure physical activity energy expenditure in real-world settings. One validation study found that the wearable underestimates step counts in patients with arthritis.  Reference: https://academic.oup.com/ptj/article/96/7/1093/2864929 | No peer-reviewed literature is available on ActivPAL cybersecurity specific to clinical monitoring. |
| Omron Walking Style II (HJ-720IT) | Yes | Yes | Growing evidence supports the validity of the research-grade SenseWear Armband to measure physical activity in COPD patients in real-world settings. One validation study with the device in real-world settings with COPD patients found that the wearable accurately records step counts in real-world settings  Reference: https://www.proquest.com/docview/1696888918?pq-origsite=gscholar&fromopenview=true&sourcetype=Scholarly%20Journals | OMRON explicitly states it does not collect geographic location data. No peer-reviewed literature is available on OMRON cybersecurity specific to clinical monitoring. |
| Belt-worn 3D-accelerometer MTx-W sensor | No | No | Little evidence supports the validity of the research-grade MTx-W sensor to measure step counts in COPD patients in real-world settings. No validation studies with the wearable exist. | The Xsens MTx-W sensor is no longer supported by Movella and has no software updates available. No peer-reviewed literature is available on Xsens MTx-W sensor cybersecurity specific to clinical monitoring. |
